# Guillain-Barré syndrome following SARS-CoV-2 vaccination in the UK: a prospective surveillance study

**DOI:** 10.1136/bmjno-2022-000309

**Published:** 2022-07-12

**Authors:** Arina A Tamborska, Bhagteshwar Singh, Sonja E Leonhard, Eva Maria Hodel, Julia Stowe, Taylor Watson-Fargie, Peter M Fernandes, Andreas C Themistocleous, Jacob Roelofs, Kathryn Brennan, Caroline Morrice, Benedict D Michael, Bart C Jacobs, Helen McDonald, Tom Solomon, UK Covid Vaccine GBS Study Group, Christopher M Allen

**Affiliations:** 1 National Institute for Health Research Health Protection Research Unit for Emerging and Zoonotic Infections, University of Liverpool, Liverpool, UK; 2 Department of Neurology, The Walton Centre NHS Foundation Trust, Liverpool, UK; 3 Tropical and Infectious Diseases Unit, Royal Liverpool University Hospital, Liverpool, UK; 4 Department of Neurology, Erasmus MC, Rotterdam, the Netherlands; 5 Institute of Infection, Veterinary and Ecological Sciences, University of Liverpool, Liverpool, UK; 6 UK Health Security Agency, London, UK; 7 Institute of Neurological Sciences, Queen Elizabeth University Hospital, Glasgow, UK; 8 Department of Clinical Neurosciences, Royal Infirmary of Edinburgh, Edinburgh, UK; 9 Nuffield Department of Clinical Neurociences, University of Oxford, Oxford, UK; 10 Neurosciences Centre, Royal Victoria Infirmary, Newcastle upon Tyne, UK; 11 GAIN (Guillain-Barré & Associated Inflammatory Neuropathies) Charity, Sleaford, UK; 12 Department of Neurology and Immunology, Erasmus MC, Rotterdam, The Netherlands; 13 NIHR Health Protection Research Unit in Vaccines and Immunisation, London School of Hygiene & Tropical Medicine, London, UK

**Keywords:** clinical neurology, guillain-barre syndrome, COVID-19

## Abstract

**Objective:**

To investigate features of Guillain-Barré syndrome (GBS) following SARS-CoV-2 vaccines and evaluate for a causal link between the two.

**Methods:**

We captured cases of GBS after SARS-CoV-2 vaccination through a national, open-access, online surveillance system. For each case, the certainty of GBS was graded using the Brighton criteria, and the relationship to the vaccine was examined using modified WHO Causality Assessment criteria. We compared age distribution of cases with that of prepandemic GBS cases and clinical features with the International GBS Outcome Study (IGOS).

**Results:**

Between 1 January and 30 June 2021, we received 67 reports of GBS following the ChAdOx1 vaccine (65 first doses) and three reports following the BNT162b2 vaccine (all first doses). The causal association with the vaccine was classified as probable for 56 (80%, all ChAdOx1), possible for 12 (17%, 10 ChAdOx1) and unlikely for two (3%, 1 ChAdOx1). A greater proportion of cases occurred in the 50–59 age group in comparison with prepandemic GBS. Most common clinical variants were sensorimotor GBS (n=55; 79%) and facial diplegia with paraesthesias (n=10; 14%). 10% (n=7/69) of patients reported an antecedent infection, compared with 77% (n=502/652) of the IGOS cohort (p<0.00001). Facial weakness (63% (n=44/70) vs 36% (n=220/620); p<0.00001) and sensory dysfunction (93% (n=63/68) vs 69% (n=408/588); p=0.00005) were more common but disease severity and outcomes were similar to the IGOS study.

**Interpretation:**

Most reports of GBS followed the first dose of ChAdOx1 vaccine. While our study cannot confirm or refute causation, this observation, together with the absence of alternative aetiologies, different than expected age distribution and the presence of unusual clinical features support a causal link. Clinicians and surveillance bodies should remain vigilant to the possibility of this very rare adverse event and its atypical variants.

**What is already known on this topic**

- Evidence is accumulating that adenovirus-vectored vaccines may cause Guillain-Barré syndrome (GBS), but little is known about the clinical features and outcomes of patients affected by this very rare adverse event

**What this study adds**

- Based on the cohort of 70 UK patients who developed GBS following SARS-CoV-2 vaccination, we show that while clinical features may differ, the outcomes and mortality are similar to prepandemic GBS.

**How this study might affect research, practice or policy**

- With billions of people yet to be vaccinated, this highlights the importance of ongoing vigilance and provides reassurance about the rarity of the event and the outcomes of those affected.

## Introduction

Vaccination against SARS-CoV-2 is the most important public health measure to control the COVID-19 pandemic. In the UK, the vaccination programme began on the 8th of December 2020 with frontline health and social care staff, care home residents, and the elderly and then cascaded down through other priority groups so that, a year later, over 50 million people received at least one dose of one of the three vaccines: BNT162b2 (Pfizer- BioNTech) mRNA vaccine, ChAdOx1 (Oxford-AstraZeneca) adenovirus-vectored vaccine or the mRNA-1273 (Moderna) vaccine.^1 2^ Monitoring for adverse event is a critical part of any vaccination programme. In the UK, health professionals and the public report suspected adverse events to the UK Medicines & Healthcare products Regulatory Agency (MHRA) via the online ‘Yellow Card’ system. In addition, based on knowledge of previous vaccines, adverse events of special interest are designated for focused evaluation by the MHRA. In January 2021, to support the monitoring work of the MHRA, we set up a national notification system for neurological adverse events of special interest, including Guillain-Barré syndrome (GBS).

GBS is a rare, acute autoimmune inflammatory polyradiculoneuropathy. The risk of GBS increases with age; the overall incidence before the COVID-19 pandemic was estimated at 1.1 to 1.8 per 100 000 per year.^3^ The most common form of GBS, acute inflammatory demyelinating polyneuropathy (AIDP), is characterised by an areflexic, symmetrical, flaccid quadriparesis, often with sensory, autonomic and cranial nerve dysfunction and, in severe cases, respiratory failure.^4^ About two-thirds of patients with GBS have symptoms of an infection in the preceding 6 weeks, which is thought to trigger the immune response against the peripheral nervous system. Although many pathogens have been implicated, the association with GBS has been confirmed just for six in case–control studies: *Campylobacter jejuni*, cytomegalovirus, hepatitis E virus, *Mycoplasma pneumoniae*, Epstein-Barr virus and Zika virus.^4^ Whether SARS-CoV-2 is a trigger for GBS remains uncertain.^5–8^ Some vaccines have also been linked to GBS, particularly influenza vaccines.^9 10^

To date, there has been a handful of publications of GBS following SARS-CoV-2 vaccination.^11 12^ On 9 July 2021, the European Medicines Agency recommended a change to the product information for CHAdOx1 (marketed as Vaxzevria in the EU) to raise awareness of this and subsequently assessed the causal relationship between GBS and ChAdOx1 as a ‘reasonable possibility’.^13 14^ Similarly, on 13 July, the US Food and Drug Administration issued a warning of an increased risk of GBS after the adenovirus-vectored Ad26.COV2-S vaccine (Janssen) and subsequently estimated the rate of GBS reports as 20.2 per million doses administered (95% CI 8.1 to 41.7).^15 16^

Through our national UK surveillance study, we aimed to capture clinical and demographic information on the cases of GBS following SARS-CoV-2 vaccines and to evaluate the probability of a causal link between the two. Our study contributes to the growing evidence on the subject, while describing the patient demographics, clinical features, progression and outcomes in comparison with the prepandemic GBS.

## Methods

### Study design and participants

We set up a UK-wide, open-access, online system for any clinician to report neurological adverse events following any SARS-CoV-2 vaccination via the Association of British Neurologists’ Rare Diseases Ascertainment and Recruitment programme.^17^ This was supplemented by direct email communication with neurology colleagues nationwide. We did not restrict the time window for a possible link between a vaccine and an adverse event. This surveillance study, using routine patient data in anonymised form, could proceed without patient consent or review by an ethics committee, as per The UK Health Research Authority guidance.^18^

### Procedures

On submitting a notification, a clinician received a standardised reporting form, collecting details on patient’s demographics, medical background, vaccination, clinical features, investigations and outcomes (appendix). This was developed based on the Brighton Collaboration recommendations for assessing postvaccination GBS^19^ and the WHO Causality Assessment of an Adverse Event Following Immunisation (AEFI),^20^ in consultation with the MHRA. The same form was also sent to those reporting potential GBS cases directly to the MHRA via the Yellow Card system. The form is freely available for downloading via our website,^21^ together with forms for other neurological adverse events.

Completed case records were submitted, stored and analysed in accordance with the general data protection regulations. Each case was ascertained and causality assessed by two independent clinicians with disagreements resolved via discussion with a third assessor, if necessary.

For patients presenting with limb weakness (the most common presentation of GBS) or with Miller Fisher syndrome, we assessed the level of diagnostic certainty using the Brighton Collaboration criteria^19^; these classify cases from level 3 (a clinical picture consistent with GBS) to level 1 (a clinical picture and confirmatory diagnostic testing consistent with GBS) (table 1). As the Brighton criteria do not apply to all variants of GBS, we included atypical forms, as have others.^4 22 23^ These were confirmed to be consistent with GBS by independent panel of dedicated GBS researchers and graded as ‘level 4’ of diagnostic certainty, as proposed previously for GBS following COVID-19.^24^ For the facial diplegia with paraesthesias variant, we graded the level of diagnostic certainty using a modified version of the Brighton criteria (table 1).

**Table 1:** Overview of Brighton Collaboration criteria for diagnosis of Guillain-Barré syndrome (GBS) and Miller Fisher syndrome and proposed criteria for diagnosis of facial diplegia with paraesthesias variant of GBS, adapted from the Brighton Collaboration criteria^19 24^

GBS variant	Level of diagnostic certainty			
	Level 1	Level 2	Level 3	Level 4
Sensorimotor and motor variants	Bilateral and flaccid weakness of the limbs AND Decreased or absent deep tendon reflexes in weak limbs AND Monophasic illness with weakness nadir 12 hours to 28 days from the onset and subsequent clinical plateau AND Absence of an alternative diagnosis			Suspected GBS with no other diagnosis apparent, which does not meet level 3 criteria
	CSF white cell count <50 cells/µL OR If CSF unavailable, electrophysiological studies consistent with GBS		-	
	CSF white cell count <50 cells/µL with raised CSF protein AND Electrophysiological studies consistent with GBS	-		

	Level 1	Level 2	Level 3	Level 4
Miller Fisher Syndrome	Bilateral ophthalmoparesis AND Bilateral decreased or absent tendon reflexes AND Ataxia AND Monophasic illness with weakness nadir 12 hours to 28 days from the onset and subsequent clinical plateau AND Absence of limb weakness AND Absence of alterations in consciousness or corticospinal tract signs AND Absence of an alternative diagnosis			Suspected Miller Fisher syndrome with no other diagnosis apparent, which does not meet level 3 criteria
	CSF white cell count <50 cells/µL OR Nerve conduction studies normal OR show involvement of sensory nerves only		-	
	CSF white cell count <50 cells/µL with raised CSF protein AND Nerve conduction studies normal OR show involvement of sensory nerves only	-		

	Level 1	Level 2	Level 3	Level 4
Facial diplegia with paraesthesias	Bilateral lower motor neuron facial weakness AND Bilateral paraesthesia of the lower and/or upper limbs AND Absence of limb weakness AND Monophasic illness with weakness nadir 12 hours to 28 days from the onset and subsequent clinical plateau AND Absence of an alternative diagnosis			Suspected facial diplegia with paraesthesias variant with no other diagnosis apparent, which does not meet level 3 criteria
	CSF white cell count <50 cells/µL OR If CSF unavailable, electrophysiological studies consistent with GBS		-	
	CSF white cell count <50 cells/µL with raised CSF protein AND Electrophysiological studies consistent with GBS	-		

CSF, cerebrospinal fluid.

### Patient demographics

We described the age distribution of GBS cases after SARS-CoV-2 vaccination and compared this with the age distribution of prepandemic GBS cases. Because GBS incidence estimates vary by setting,^3 11^ we obtained data for background GBS rates from Hospital Episode Statistics Admitted Patient Care data, which holds details of discharge diagnoses for all NHS hospital admissions in England.^25^ We defined an incident case of GBS as an International Classification of Diseases-10 code of G61.0 (Guillain-Barré syndrome) or G52.7 (disorders of multiple cranial nerves, to capture polyneuritis cranialis) in any of the first five diagnosis fields. Repeat episodes within 365 days for the same patient were identified by using the unique identifier Hospital Episode Statistics ID and excluded. A previous study found this approach had a high positive predictive value for an incident episode of GBS.^26^ We combined admissions between 1 January 2015 and 31 December 2019 to calculate the age distribution of GBS cases in England during this period. Age-specific incidence rates for the period were calculated using Office for National Statistics midyear population estimates from 2020.

### Clinical features and outcomes

To examine whether the clinical features of GBS following SARS-CoV-2 vaccination differed from GBS linked to other causes, we compared our patients with those recruited by the International GBS Outcome Study (IGOS), a prospective observational study published the year before the COVID-19 pandemic started.^27^ Because there are regional differences in GBS variants, treatment and outcomes between low-income and middle-income versus high-income countries,^27 28^ we compared our patients with the European and American cohort of the IGOS study consisting of 715 (77%) of the total 925 IGOS patients. Serum of our patients was tested for antiganglioside antibodies through NHS diagnostic laboratories at the discretion of the treating clinicians, and nerve conduction studies were conducted as clinically indicated using local protocols and categorised locally into demyelinating, axonal, inexcitable, equivocal or normal by the reporting clinicians.

Outcome was assessed at 3 months after vaccination using the GBS disability score at routine inpatient or outpatient follow-ups held by the treating clinicians.^29^ At this point, we also assessed patients’ clinical course and determined whether some patients initially diagnosed with GBS might have had an alternative diagnosis, including acute onset chronic inflammatory demyelinating polyneuropathy (CIDP), which is a recognised mimic of GBS early on.^30^

### Causality assessment for GBS following vaccination

To examine the putative link between vaccine and clinical presentation, we applied the WHO Causality Assessment for AEFI^20^ modified to allow for the fact that there is no previous literature confirming an association between GBS and the new SARS-CoV-2 vaccines.^31^ Rather than classifying cases as being ‘consistent’ or ‘inconsistent’ for a causal association with the vaccine, we classified them as having a ‘confirmed’, ‘probable’, ‘possible’ or ‘unlikely’ link (table 2). This is because the WHO manual requires pre-existing evidence of an association between the adverse events and the vaccine for the events to be categorised as ‘consistent’ or ‘inconsistent’ and such evidence is not available for new vaccine products. The approach we used follows the WHO-Uppsala Monitoring Centre System for Standardised Case Causality Assessment,^32^ from which WHO AEFI classification was derived, and is similar to the one we used previously to define newly described neurological associations with COVID-19.^24^

**Table 2:** Criteria for causality assessment for Guillain-Barré syndrome (GBS) in temporal association with vaccination

Proposed causality label	Generic assessment criteria based on WHO Causality Assessment^20^	Proposed GBS specific causality assessment criteria
Confirmed	Published peer-reviewed epidemiological evidence supporting causative association with the vaccination AND Typical time frame AND No indication of another cause for the event AND No illness, pre-existing condition or risk factors that could have contributed to the event, as excluded by detailed history, clinical examination and investigations	Administration of a vaccine confirmed to increase risk of GBS AND Event ≥24 hours and ≤6 weeks from vaccination^19^ AND No indication of an alternative aetiology, including symptoms of infectious illness in the preceding 6 weeks* as excluded by detailed history, clinical examination and investigations
Probable	Typical time frame AND No indication of another cause for the event AND No illness, pre-existing condition or risk factor that could have contributed to the event, as excluded by detailed history, clinical examination and investigations	Event ≥24 hours and ≤6 weeks from vaccination^19^ AND No indication of an alternative aetiology, including symptoms of infectious illness in the preceding 6 weeks* as excluded by detailed history, clinical examination and investigations
Possible	Plausible time frame but outside of typical OR There may be an indication of another cause, predisposing condition and/or risk factors, but these are unlikely to fully explain the event	Event >6 weeks and <12 weeks from vaccination^19^ OR Clinical or microbiological evidence of an infection, but not one of those which has a proven link to GBS (listed further), or a physiological stress, in the 6 weeks preceding the event
Unlikely	Timeframe not fitting with the event OR Evidence of another cause, predisposing condition and/or risk factors that fully explain the event	Event <24 hours or >12 weeks from vaccination^19^ OR Clinical suspicion or microbiological evidence of infection with: *Campylobacter jejuni*, influenza, Cytomegalovirus, Epstein–Barr virus, *Mycoplasma pneumoniae*, *Haemophilus influenzae*, hepatitis E, or Zika virus in a typical timeframe; or treatment with tumour necrosis factor antagonist, immune-checkpoint inhibitor or type I interferon^4 22 53^

*This excludes symptoms of reactogenicity such as fever, myalgia and fatigue in the first 72 hours after vaccination.^54^ While most antecedent infections precede GBS by 4 weeks, a longer cut-off was selected based on the previous reports of GBS occurring up to 6 weeks post-influenza infection.^55^

### Statistical analysis

We compared descriptive statistics for patients with GBS following SARS-CoV-2 vaccination, with the Europe/Americas cohort from the IGOS study. We compared categorical variables between groups using χ^2^ test, or Fisher’s exact test if the expected number of patients in any one category was less than five. We set statistical significance at 5%. To examine the robustness of our findings, we performed a sensitivity analysis that included only the subgroup of patients in whom the diagnosis of GBS was most certain, that is, Brighton criteria levels 1 or 2. In another sensitivity analysis, we included only the patients with the strongest link between GBS and the SARS-CoV-2 vaccination (those classified as ‘probable’ by the modified WHO Causality Assessment Criteria). We used Microsoft Excel and IBM SPSS software (V.27, IBM Corp, Armonk, New York, USA).

### Role of the funding source

No funding body had a role in the study design, data collection, analyses, interpretation or writing of the article. The corresponding author had full access to all data and final responsibility for the decision to publish.

## Results

### Number of reports

**Figure 1:**
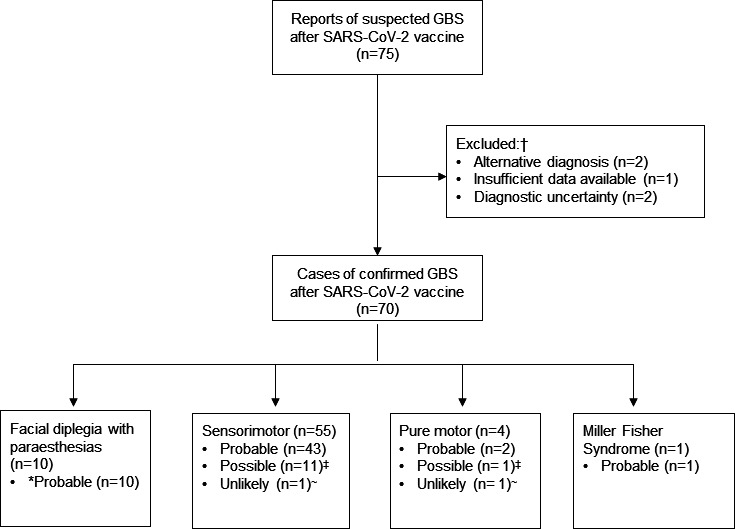
Study flow chart showing clinical variants of Guillain-Barré syndrome (GBS) and levels of certainty of a link to the vaccine, as determined by the modified WHO Causality Assessment. *Levels of certainty of a link to the vaccine, as determined by the modified WHO Causality Assessment.^20^ †The five excluded cases comprised one with insufficient data, two with alternative diagnosis (neurosarcoidois and CIDP) made subsequently by the treating clinicians and two rapidly fatal cases that had features inconsistent with GBS and were excluded following discussions with the independent assessors. One of these two cases had upgoing plantars, normal CSF protein and no imaging or nerve conduction studies performed prior to death. The second case had normal CSF protein but raised white cell count and widespread demyelination on brain and spinal MRI. ‡Reasons for categorisation as ‘possible’ (n=12) included: antecedent infection without a recognised microbiological trigger for GBS ((n=5): URTI (n=2), IECOPD (n=1), Klebsiella urinary tract infection (n=1), gastroenteritis with no suspicion of Campylobacter and in unlikely temporal association with GBS (n=1)),^53^ raised C reactive protein without infective symptoms (n=1), use of small molecule inhibitors anecdotally associated with GBS (n=1),^56^ presence of systemic disease that might cause GBS-mimicking neuropathy ((n=3): CLL with acute axonal neuropathy (n=1)^57^ and MGUS with AIDP (n=1),^58^ suspected endocrinopathy and functional neurological overlay (n=1)), overlay with subacute demyelinating neuropathy (n=1), event occurring between 6 and 12 weeks from vaccination (n=1). ~Reasons for categorisation as ‘unlikely’ included microbiological evidence or clinical suspicion of *Campylobacter jejuni* infection (n=2). AIDP, acute inflammatory demyelinating polyradiculoneuropathy; CIDP, chronic inflammatory demyelinating polyneuropathy; CLL, chronic lymphocytic leukaemia; CSF, cerebrospinal fluid; IECOPD, infective exacerbation of chronic obstructive pulmonary disease; MGUS, monoclonal gammopathy of undetermined significance; URTI, upper respiratory tract infection.

**Figure 2:**
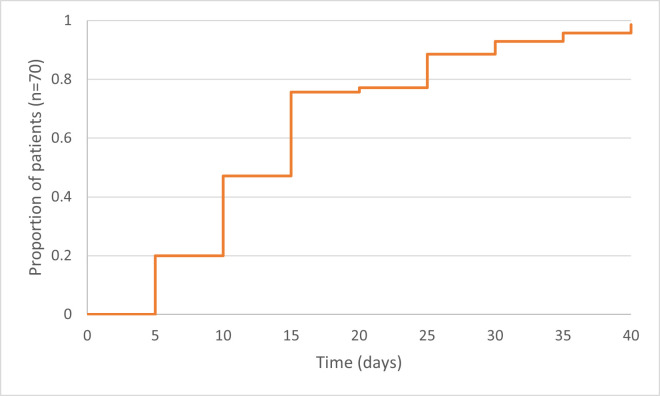
Time from SARS-CoV-2 vaccination to the onset of Guillain-Barré syndrome. a Median time from vaccination to symptom onset was 15 days (IQR 10–19). fTwo out of seventy subjects developed symptoms after more than 6 weeks (46 and 55 days).

Between 1 January and 30 June 2021, we received 75 reports of GBS following SARS-CoV-2 vaccination (including seven cases reported previously),^11 33 34^ of which 70 were included in the study (figure 1). Sixty-seven (96%) of the 70 had received the ChAdOx1 vaccine and three (4%) received the BNT162b2 vaccine, a median 15 (IQR 10–19) days before the first symptoms of GBS (figure 2). All but two (both ChAdOx1) were first doses. At the same time, an estimated 30 million doses (19 million first doses) of BNT162b2, 46 million doses (25 million first doses) of ChAdOx1 and 1 million single doses of mRNA-1273 were administered in the UK.

For 67 (96%) of the 70, the case record form was completed by a neurology or neurophysiology consultant or specialty trainee. Forty-six (66%) were from tertiary centres and 24 (34%) from district general hospitals from all seven regions of NHS England and from Scotland and Wales. The earliest vaccination was on 15 January 2021 and the latest 23 April. The earliest onset of GBS was 1 February 2021 and the latest 14 May. In the same time window, the MHRA received 407 Yellow Card notifications of ‘acute polyneuropathies’ after SARS-CoV-2 vaccination from healthcare professionals and members of the public.^35^

The 70 patients with GBS following SARS-CoV-2 vaccination comprised 55 (79%) with classic sensorimotor GBS, 4 (6%) pure motor GBS, 10 (14%) facial diplegia with paraesthesias and 1 (1%) Miller Fisher syndrome (table 3). This distribution differed from that for the IGOS cohort in which 69% had sensorimotor, 14% pure motor GBS, 11% MFS and 6% other variants (p=0.0012 for difference in the distribution of clinical variants, table 3).^27^ Thirty-three (47%) of the 70 met Brighton criteria level 1, and 28 (40%) met Brighton criteria level 2; 9 of 10 patients who had facial diplegia with paraesthesias met level 1 or 2 for a diagnosis of GBS according to the modified Brighton criteria (table 1). Nine patients (13%) were diagnosed clinically with GBS and had investigation results consistent with GBS but did not meet other criteria for Brighton levels 1–3. There were no patients in the level 3 category.

**Table 3:** Comparison of patients with Guillain-Barré syndrome (GBS) following SARS-CoV-2 vaccination and previously published European or American patients from the International GBS Outcome Study (IGOS)

	GBS after SARS-CoV-2 vaccination (n=70)*	IGOS Europe and Americas cohort (n=715)	Statistical comparison (p value)
**Patient characteristics**			
Sex at birth			
Female	34/70 (49%)	297/715 (42%)	0.26
Male	36/70 (51%)	418/715 (58%)	
Age			
Mean (SD)	57.84 (±13.05)	53 (±22.28)†	0.07
Ethnicity			
Asian	1/70 (1%)	NA	NA
White	69/70 (99%)		
Reported antecedent infection (6 weeks prior)			
Yes	7/69 (10%)	502/652 (77%)	<0.0001
**Clinical features**			
Clinical variant			
Sensorimotor	55/70 (79%)	388/562 (69%)	0.0012
Pure motor	4/70 (6%)	78/562 (14%)	
MFS	1/70 (1%)	62/562 (11%)	
Other‡	10/70 (14%)	34/562 (6%)	
Sensory dysfunction§			
Yes	63/68 (93%)	408/588 (69%)	<0.0001
Oculomotor weakness			
Yes	9/66 (14%)	84/620 (14%)	0.87
Facial weakness			
Yes	44/70 (63%)	220/620 (36%)	<0.0001
Bulbar weakness			
Yes	17/65 (26%)	136/620 (22%)	0.54
Autonomic dysfunction			
Yes	16/67 (38%)	184/626 (29%)	0.34
Pain			
Yes	29/70 (41%)	354/625 (57%)	0.0153
Shortness of breath			
Yes	14/70 (20%)	NA	
Time from onset to nadir			
Median (IQR)	11 days (7 - 15)	NA	
Unable to walk independently at nadir			
Yes	51/70 (73%)	478/626 (76%)	0.42
Ventilator dependency			
Yes	7/70 (10%)	121/715 (17%)	0.14
**Investigations**			
Antiganglioside antibodies¶			
Positive/tested	1/49 (2%)	NA	NA
Electrophysiological variant**			
Demyelinating	46/54 (85%)	312/573 (55%)	<0.0001
Axonal	3/54 (6%)	33/573 (6%)	
Inexcitable	0/54 (0%)	10/573 (2%)	
Equivocal	1/54 (2%)	182/573 (32%)	
Normal	4/54 (8%)	36/573 (6%)	
**Initial treatment††**			
None	11/70 (16%)	54/715 (7%)	0.0180
IVIG	50/68 (74%)	612/661 (93%)	<0.0001
PLEX	0/68 (0%)	43/661 (6%)	
Other	7/68 (10%)	6/661 (1%)	

*31 of our patients had chronic comorbidities, including hypertension (n=15), depressive disorder (n=7), chronic respiratory disease, including asthma (n=6), thyroid disease (n=6) and diabetes mellitus (n=5); some patients had more than one comorbidity.

†Mean age and its SD for IGOS cohort was derived from median and IQR, as described previously.^59^

‡Other clinical variants included bilateral facial diplegia with paraesthesias variant in our cohort and pharyngo-cervical-brachial, pure sensory, ataxic or other variants in IGOS cohort.

§Sensory dysfunction excludes pain.

¶Antiganglioside antibody testing panel for most patients (40 (82%) of 49) included GM1 IgG, GM2 IgG, GD1a IgG, GD1b IgG, GQ1b IgG, GM1 IgM, GM2 IgM, GD1a IgM, GD1b IgM and GQ1b IgM.

**54 patients in our study had nerve conduction studies performed and results available, of whom 32 also had electromyography. The median time to electrophysiological studies was 15 days (IQR 11–25.5; data available for 47 patients patients). Ninety per cent of patients had electrophysiological studies performed at least 1 week from the symptom onset. Like IGOS, we report here the first electrophysiology results, accepting that axonal degeneration may only become manifest at a later time, and that if NCS/EMG is repeated after several weeks some patients need to be reclassified electrophysiologically. The diagnoses for our patients are those given by the reporting clinician, whereas for IGOS, the raw data were analysed centrally according to criteria of Hadden *et al*.^36^

††Data on first-line treatment given was available for 68 of the patients who had GBS following SARS-CoV-2 vaccination. The ‘other’ treatment category comprised five patients who initially received corticosteroids and two who had IVIG and corticosteroids together. In the IGOS cohort, ‘other’ included corticosteroids, immunoadsorption and trial medication. Three IVIG recipients in our study had plasma exchange subsequently; two of whom then received a further course of IVIG.

IVIG, intravenous immunoglobulin; MFS, Miller Fisher Syndrome; PLEX, plasma exchange.

### Patient demographics

**Figure 3:**
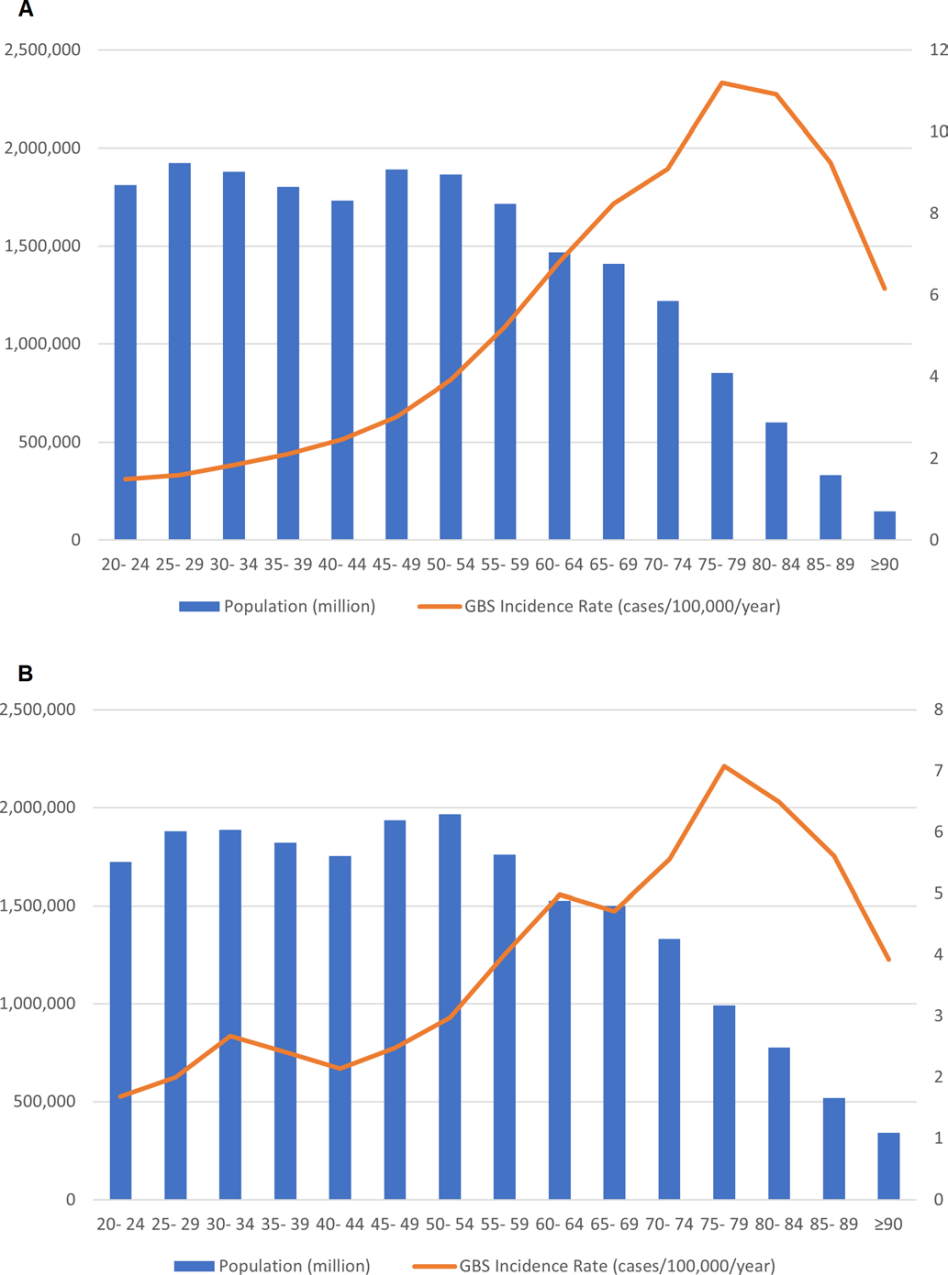
Population (left-hand axis) and background Guillain-Barré syndrome (GBS) incidence rate (right-hand axis) -among adult men (A) and women (B) in England, 2015–2019, per age group.

**Figure 4:**
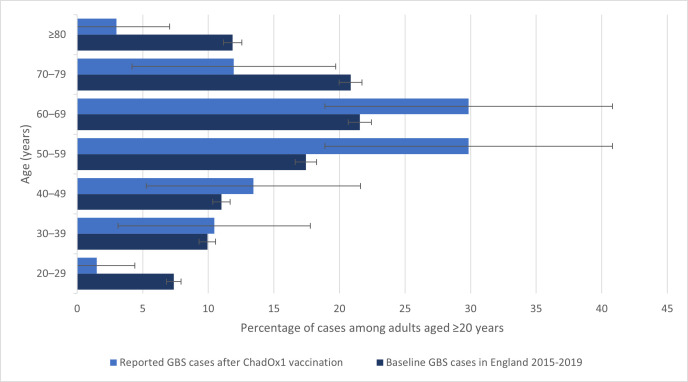
Age distribution of GBS cases reported after ChAdOx1 vaccination (n=67) compared with background GBS cases in adults in England 2015–2019 (n=8423)*. *Background incident cases of GBS were identified from Hospital Episode Statistics Admitted Patient Care data, as described previously,^26^ and the age-specific incidence rates were calculated using Office for National Statistics midyear population estimates from 2020. The proportion of postvaccination GBS cases occurring at ages 50–59 years was higher than among baseline cases (29.9%, 95% CI 18.9 to 40.8 vs baseline 17.4%, 95% CI 16.6 to 18.3), while the proportion of postvaccination cases was lower than baseline for ages 20–29 years (1.5, 0 to 4.4 vs 7.4, 6.8 to 7.9), 70–79 years (11.9, 4.2 to 19.7 vs 20.8, 20.0 to 21.7) ≥80 years (3.0, 0 to 7.1 vs 11.9, 11.2 to 12.6). GBS, Guillain-Barré syndrome.

The median age of patients with GBS following SARS-CoV-2 vaccination was 59 (IQR 51–67) years, 36 (51%) were men, and all but one were white (table 3). The background incidence (cases per 100 000) for GBS in the UK population prepandemic increased steadily with age up to 75 years and then declined (figure 3), consistent with previous studies.^3^ The background age distribution of GBS cases in the UK 2015–2019 (ie, the proportion of cases in each age category) also increased with age up to 75 years and then declined, whereas the age distribution of reported cases of GBS after ChadOx1 vaccination was different (p=0.005 for the difference in distribution; figure 4). It was higher at ages 50–59 years and lower at ages 20–29 and ≥80 in comparison with the prepandemic GBS. The age distribution for GBS after the BNT162b2 vaccine was not calculated because there were only three cases.

Thirty-one (45%) of 69 patients for whom the information was available had an underlying medical condition, including six (9%) with a neurological disorder. Four (6%) of 70 were immunocompromised: two on corticosteroids, one on immunotherapy for malignant myeloma and one with a concurrent diagnosis of chronic lymphocytic leukaemia. One patient had had GBS as a child following the measles–mumps–rubella vaccine. None of 65 patients tested had COVID-19 at presentation, but 3 of 55 with data available had been diagnosed with COVID-19, 3, 4 and 12 months before vaccination.

### Clinical features

The most common presentation after SARS-CoV-2 vaccination was a sensorimotor GBS with flaccid, areflexic quadriparesis, often with sensory dysfunction and cranial nerve involvement, causing an inability to mobilise (table 3). Forty-seven (67%) patients had quadriparesis, 10 (14%) paraparesis, 2 (3%) other pattern of weakness (one unilateral and one upper limbs only) and 11 (16%) no limb weakness (10 with facial diplegia and paraesthesias, 1 with Miller Fisher syndrome); this pattern was similar to that in the IGOS study.^27^ However, patients with GBS after SARS-CoV-2 vaccination were more likely than those in the IGOS cohort to have sensory dysfunction and to have facial weakness. These observations remained true in a sensitivity analysis that included only the 61 patients meeting Brighton criteria levels 1–2 and another that included only the 56 patients where the link to SARS-CoV-2 vaccination was classified as ‘probable’ by the modified WHO Causality Assessment Criteria. Sixty-three patients underwent lumbar puncture, a median 11 (IQR 6–17) days from symptom onset, of whom 62 had the cerebrospinal fluid total protein evaluated. This was elevated in 59; the other three had their lumbar punctures at 1, 18 and 43 days from symptom onset.

### Progress and outcome

The median time to reach the nadir of maximum weakness for GBS after SARS-CoV-2 vaccination was 11 days (IQR 7–15); seven (10%) patients required ventilation. The disease severity, as judged by the number unable to walk independently at the nadir, was similar to that of the IGOS cohort (table 3). This remained true after exclusion of patients who had facial diplegia with paraesthesias, which tend to be milder. However, fewer patients with GBS after COVID-19 vaccination received treatment than in the IGOS cohort; this was accounted for by the greater proportion who had facial diplegia with paraesthesias, which often is not treated. Forty-nine patients were tested for antiganglioside antibodies; one, with classic sensorimotor AIDP after SARS-CoV-2 vaccination, was positive for serum anti-GQ1b antibodies. On nerve conduction studies, the main subtype for patients with GBS following SARS-CoV-2 vaccination was similar to that for IGOS patients, with demyelinating disease dominating over axonal disease (table 3); however, in IGOS, where results were categorised centrally according to exacting electrophysiological criteria,^36^ more patients were classed as equivocal.

Four (6%) patients required only outpatient care, but the majority (94%; n=66) were hospitalised, including 6 (9%) managed on the high dependency unit, and 10 (14%) who needed intensive care (table 4). Two (3%) patients died. One death was due to hospital-acquired pneumonia 61 days after symptom onset; the other was a sudden cardiac arrest at 29 days, attributed at postmortem to a deep vein thrombosis with secondary pulmonary embolism. At data lock 46 (66%) patients had been discharged home, and 22 (31%) were in hospital, including 13 (18%) in rehabilitation.

**Table 4:** Outcomes for patients with Guillain-Barré syndrome (GBS) following SARS-CoV-2 vaccination

Outcome	GBS after SARS-CoV-2 vaccination (n=70)
Maximum level of care	
Outpatient	4/70 (6%)
Medical ward	50/70 (71%)
High dependency unit	6/70 (9%)
Intensive care unit	10/70 (14%)
Discharge destination	
Usual place of residence	46/70 (66%)
Medical ward	9/70 (13%)
Rehabilitation	13/70 (18%)
Died during admission	2/70 (3%)
Duration of admission	
Median (IQR)	13.5 days (8–28.5; for n=52)
GBS disability score at 3 months	
No symptoms (score 0)	5/59 (9%)
Symptomatic but able to run (score 1)	20/59 (34%)
Able to walk independently, but unable to run (score 2)	9/59 (15%)
Mobilising with aids (score 3)	13/59 (15%)
Wheelchair bound or bedbound (score 4)	9/59 (15%)
Ventilated for at least a part of the day (score 5)	1/59 (2%)
Died (score 6)	2/59 (3%)
Further SARS-CoV-2 vaccination	
Yes	7/70 (10%)*

Further three patients (two with sensorimotor AIDP and one with facial diplegia with paraesthesias variant) who initially received ChAdOx1 opted to receive BNT162b2 for their second dose. For all, the GBS was classified as probably linked with the vaccine. None had any new symptoms or deterioration.

*Four patients (three with classic sensorimotor AIDP and one with facial diplegia with paraesthesias variant) received a second dose of the same SARS-CoV-2 vaccine after their acute illness. One, whose GBS had been classified as unlikely linked to the vaccine, had a further dose of BNT162b2; one, whose GBS was possibly linked to the vaccine, had ChAdOx1; and two, whose GBS was classified as probably linked, had a further dose of ChAdOx1. None had any new symptoms or deterioration.

AIDP, acute inflammatory demyelinating polyneuropathy.;

The median follow-up was 83 (IQR 61–105) days; all but one patient were followed up for at least 4 weeks and 93% (n=65) for at least 6 weeks. The GBS disability score was assessed at 3 months for 59 patients (table 4). Four (7%) patients were considered at this stage to have had possible acute onset CIDP; two who had had only mild disease and no cranial nerve involvement, one who subsequently developed optic neuritis and one with an indolent disease course and steroid treatment related fluctuations. These patients were still thought to have GBS by the treating clinicians, whereas the patients who were ultimately diagnosed with CIDP (n=1) were excluded from the study cohort (figure 1).

Four patients (three with classic sensorimotor AIDP and one with the facial diplegia with paraesthesias variant) received a second dose of the same SARS-CoV-2 vaccine after their acute illness, three with ChAdOx1 and one BNT162b2; in addition, three patients (two with sensorimotor AIDP and one with facial diplegia with paraesthesias) who initially received ChAdOx1 had BNT162b2 for their second dose. None of these patients had any new symptoms or deterioration following their second vaccination. Completion of vaccination schedule with BNT162b2 vaccine has since become recommended in the UK in individuals who developed GBS following ChAdOx1 vaccination.^37^

### Causality assessment

Seven (10%) of 69 patients with GBS after SARS-CoV-2 vaccination reported an infection in the preceding 6 weeks, compared with 502 (77%) of 652 in the IGOS cohort (p<0.00001). Fifty-six (80%) of 70 patients had GBS within a typical timeframe (within 6 weeks) after vaccination (all ChAdOx1) and had no other causes found; for them, the association between SARS-CoV-2 vaccination and GBS was classified as probable by the modified WHO Causality Assessment criteria (figure 1). For 12 (17%) patients (including two with BNT162b2 vaccine), the association was classified as possible, either because the timeframe was plausible but not typical (n=1), or because there was another possible cause for GBS (such as infection or medication; n=7) or a GBS-mimicking neuropathy (n=4); for two patients (one BNT162b2 vaccine) with a proven alternative causes (*Campylobacter jejuni* infection), the association with the SARS-CoV-2 vaccine was classified as unlikely.

## Discussion

In this national survey, 70 cases of GBS following SARS-CoV-2 vaccination were reported by UK clinicians between January and June 2021. Sixty-seven followed the adenovirus-vectored ChAdOx1 vaccine (Oxford-AstraZeneca), of which most (56) were classified as probably linked to the vaccine by the modified WHO Causality Assessment; in contrast, just three GBS cases followed the mRNA BNT162b2 vaccine (Pfizer), none of which was classified as probably linked. The modified WHO Causality Assessment examines the timeframe between the vaccine and the adverse event, investigates for other causes, looks for known risk factors and considers previous literature of a causal link.^20^ If all these are met, the link between the vaccine and the adverse event is classified as confirmed. Because at the time of our study there was only very limited literature on GBS after SARS-CoV-2 vaccination, and no epidemiological studies, no cases could be considered as confirmed.^20^ Since then, a large epidemiological study has shown an increased risk ratio of 2.9 in the 15–21 days after vaccination with the ChAdOx1 vaccine.^8^

The rapid development and introduction of SARS-CoV-2 vaccines has prevented millions of infections and thousands of deaths around the world.^38 39^ ChAdOx1 is the most widely used SARS-CoV-2 vaccine, currently in 181 countries,^40^ with more than three billion doses ordered worldwide.^41^ Critical to vaccine introduction is postmarketing surveillance, which identifies adverse events that are too rare to be detected in clinical trials. This can include discovery of completely new syndromes, such as vaccine-induced thrombosis and thrombocytopaenia,^42^ and those such as GBS that might be anticipated based on knowledge of other vaccines.^9^

Nearly 90% of the 70 GBS cases in our study met the modified Brighton criteria levels 1 or 2 for diagnosing GBS, meaning there was a consistent clinical picture and supporting evidence from cerebrospinal fluid evaluation, nerve conduction studies or both. However, because many authorities now recognise that the Brighton criteria, published in 2011, do not include all the variant forms of GBS,^4 22 23^ we also included cases with other recognised variants. The IGOS study with which we compared also included such cases. IGOS did not apply the Brighton criteria because they miss important cases; in contrast, we modified the criteria to allow for such cases. We chose to compare with IGOS because it is the largest prospective study of GBS to date. The clinical pattern for patients with GBS after SARS-CoV-2 vaccination differed from that of the IGOS patients. Eleven per cent of our patients had the bilateral facial diplegia and paraesthesias variant of GBS; in most series, it is less than 5%.^22^ This finding carries important implications for surveillance studies: these patients, less likely to be admitted and treated with intravenous immunoglobulin, will be missed by surveillance based on hospital admission and immunoglobulin databases. Additionally, without careful evaluation, they may also mistakenly be diagnosed as having isolated facial palsy. Altogether, atypical variants accounted for nearly one-quarter of our cases and would have been missed had we relied solely on the original Brighton classification. Instead, we proposed an expansion of the Brighton criteria to incorporate such patients with facial diplegia and paraesthesias in the future.

Although the clinical presentations in our study differed from the IGOS cohort, the disease severity was similar, which is reassuring. At 3 months, the mortality was 3% in our cohort and 58% were able to mobilise independently. This is consistent with the IGOS study, where mortality at 12 months was 5%, and at 3 months just over 60% of patients were able to walk unaided.^27^ Just 10% of patients in our study recalled an antecedent infection in the preceding weeks, compared with nearly 80% in the IGOS cohort. Although this is consistent with GBS being caused by the vaccine, there are other explanations, including recall bias, or people with a current infection not getting vaccinated; however, it is unlikely these would account for the large difference we observed.

Studies of GBS following SARS-CoV-2 infection hypothesise that the viral spike protein, the key component of the ChAdOx1 vaccine, leads to formation of antiganglioside antibodies.^43^ However, in our cohort, only one patient tested positive for antiganglioside antibodies, indicating that the mechanism in vaccine-associated GBS may be different or that an antibody not routinely tested by the reporting centres may be involved. Whether the spike protein is the culprit in postvaccination GBS is also uncertain. This is because some individuals in our study suffered from SARS-CoV-2 infection with no neurological complications, yet they went on to develop GBS following SARS-CoV-2 vaccination. Similarly, few GBS cases were seen following BTN162b or mRNA-1273 vaccines that also encode the spike protein. However, both Ad26.COV2-S and ChAdOx1 vaccines use adenovirus vectors (human and chimpanzee, respectively). Yet, there is no strong evidence for adenoviruses causing GBS, and in one study, adenovirus has only been found in one of 156 GBS cases.^44^

We observed that the age distribution of GBS cases after ChAdOx1 vaccination was different to the baseline GBS age distribution before the pandemic, with a higher proportion of the postvaccination cases among adults aged 50–59 years and lower proportion among younger and older adults. There are several possible explanations, including the fact that the most elderly UK adults (aged >80) were prioritised for vaccination with BNT162b2, which was available first before ChAdOx1 became available and that the use of ChAdOx1 vaccine was restricted in the UK to the individuals older than 40 years of age.^2 45^ Alternatively, there may be ascertainment bias if GBS is less likely to be reported in the elderly, especially because of healthcare access issues during the pandemic.^46 47^ However, it is also possible that middle-aged adults may be at higher risk of GBS after ChAdOx1 vaccination than older adults. We could not undertake an observed versus expected analysis to quantify any increased risk of GBS after vaccination because the information on different vaccine usage among different age groups is not available publicly in the UK. This is important because the incidence rates vary among population as shown in figure 3, and different age groups were prioritised for different vaccine brands. However, epidemiological studies, especially self-controlled case series that are best placed to adjust for such confounders, are now addressing this. A recently published self-controlled UK case series found an increased risk of GBS from 2 weeks after a first dose of the ChAdOx1 vaccine, with an incidence rate ratio of 2.90 (95% CI 21.5 to 3.92) at 15–21 days^8^; this gave an estimated 38 excess GBS cases per 10 million vaccinees, compared with 145 excess cases per 10 million people infected with SARS-CoV-2. Interestingly, an increased risk of Bell’s palsy was also observed at 15–21 after the first dose of the ChAdOx1 vaccine (incidence ratio 1.29 (95% CI 1.08 to 1.56); it is possible that some of these patients actually had the facial diplegia with paraesthesias variant of GBS, but this had not been diagnosed as such. Our team is currently working on an independent epidemiological study to verify these findings. Consistent with our observations, other surveillance studies in the USA and in Mexico did not find increased rates of GBS after the BNT162b2 or mRNA-1273 vaccines.^48 49^ In an Israeli cohort of 579 patients with a history of GBS who received the BNT162b2 vaccine, just one had a relapse.^50^ In parallel with our study, an analysis of the intravenous immunoglobulin prescriptions in the UK identified an increase in GBS in March–April 2021, which was accounted for by the cases occurring within 6 weeks of the first dose of ChAdOx1 vaccination. Interestingly, this study did not identify phenotypical differences, although comparison was with a smaller cohort of patients who developed GBS outside of the 6 weeks temporal association with the vaccine.^51^

Our study had several limitations. Because it was a survey set up rapidly in the context of an emergency vaccination programme, we could not ensure that all patients had the same microbiological, electrophysiological and antiganglioside investigations. However, most clinicians in the UK follow the same approach to diagnosis and management.^4^ As any spontaneous surveillance system, the study is subject to a case ascertainment and reporting bias, as well as under-reporting. In adverse drug reactions surveillance, under-reporting may be as high as 94%^52^; however, serious adverse events, such as GBS, are also more likely to be reported, as may be events following SARS-CoV-2 vaccines. Fewer patients had antiganglioside antibodies than expected, possibly because antibody testing was done through routine diagnostics services rather than in a specialised laboratory. The comparison of the age-distribution of postvaccination cases to baseline GBS cases in Health Episode Statistics should be interpreted with caution, as it is limited by the different ascertainment of cases and is unadjusted for confounding, for example, by different vaccine use in different age groups.

In summary, our national study of GBS cases across the UK reported many more cases following the ChAdOx1 vaccine than the BNT162b2 vaccine; few of these patients had alternative aetiologies. Facial weakness was common with an unusually large number of patients having the facial diplegia with paraesthesias. The observed age distribution of patients with GBS after the ChAdOx1 vaccine differed from that of background GBS data before the pandemic. Mechanistic studies will be needed to examine whether in GBS after vaccination there is antibody cross-reactivity between nerve components and the adenovirus vector and/or the SARS-CoV-2 spike protein. With just 65 cases reported in our study, after 25 million first doses of ChAdOx1 vaccine, the increased risk of GBS following vaccination is likely to be very small, and the benefits of vaccination far outweigh the risks. Nevertheless, with billions of people worldwide yet to be vaccinated,^40^ the WHO Global Advisory Committee on Vaccine Safety, and medicinal product regulatory bodies will need to modify their guidance accordingly, while clinicians remain vigilant to the possibility of this rare adverse event, including its atypical variants.

10.1136/bmjno-2022-000309.supp1 Supplementary data

## Data Availability

Data are available on reasonable request. All data relevant to the study are included in the article or uploaded as supplementary information. Anonymised cumulative data can be made available for surveillance purposes on request to the corresponding author.
