# Supplementary material for: Guillain-Barré syndrome following SARS-CoV-2 vaccination in the UK: a prospective surveillance study
Source: BMJ Neurol Open. 2022 Jul 12;4(2):e000309. doi: 10.1136/bmjno-2022-000309 (PMC9277028; doi:10.1136/bmjno-2022-000309)
Supplement: Supplementary data [file bmjno-2022-000309supp001.pdf]

COVID-Neuro Network

Brain Infections Global

Suspected Guillain-Barre syndrome or peripheral neuropathy following administration of COVID-19 vaccine*

**Source of information**			
Name of the person reporting		Position (e.g. specialty and grade)	
Hospital / Practice		Email address	
Type of institution	Primary Care/ District General Hospital/ Teritiary Hospital		

**Patient Details**			
Patient Initials:		Yellow Card report reference:	
Sex:		Ethnicity:	
Age:			

**Patient Background**	
Past Medical History:	
Regular and recent medications:	
Infectious illness in the last six weeks:	Yes/ No/ Unsure
Other vaccination received in the last six weeks:	Yes/ No/ Unsure
Previous adverse neurological reaction to a vaccine:	Yes/ No/ Unsure
History of neurological disease (previous or current):	Yes/ No/ Unsure
Immunosupression at the time of vaccination:	Yes/ No/ Unsure
If Yes to any above, please provide details:	

**Patient's Covid-19 Status**	
Previous diagnosis of Covid-19:	Yes, once/ Yes, more than once/ No/ Unsure
If Yes, date of onset:	Date:
If Yes, means of diagnosis:	PCR/ Antibody / Clinical

**Vaccination Details**	
1st vaccination: Pfizer-BioNTech/ Oxford- AstraZeneca/ Moderna/ Lot number: ______ Dose: ____ Route of administration:	Date:
2nd vaccination: Pfizer-BioNTech/ Oxford- AstraZeneca/ Moderna/ Lot number: ______ Dose: ____ Route of administration:	Date:
Date of neurological symptoms onset	Date:

**Case Definition**	
For GBS: Bilateral flaccid weakness the limbs	Yes/ No/ Not Sure
PLUS Decreased or absent tendon reflexes	Yes/ No/ Not Sure
PLUS Monophasic illness with peak of symptoms 12h to 28 days from the onset and subsequent clinical plateau	Yes/ No/ Not Sure
PLUS Unknown aetiology	Yes/ No/ Not Sure

*This form, based on the guidance from the Brighton Collaboration (Sejvar et al., Vaccine 2011; 29: 599–612) and produced by the Covid-Neuro Network of the University of Liverpool's Brain Infections Global Programme, is for recording details of suspected neurological adverse events following immunisation with COVID-19 vaccines. Please feel free to modify as necessary.
Please liaise with Professor Tom Solomon and the Covid-Neuro team (Email COVIDNeuro@liverpool.ac.uk) if you are interested in contributing your data to the international collaborative study.

COVID-Neuro Network Brain Infections Global

**Clinical Features**
Time from onset to peak symptoms (hrs / days):
Limb weakness with lower motor neuron signs: Yes/ No/ Unsure<br>☐ Flaccid weakness ☐ Decreased tone ☐ Fasciculations ☐ Atrophy ☐ Other- describe<br>Affected limbs (select): RUL/ LUL/ RLL/ LLL
Sensory deficit (select these that apply): Yes/ No/ Unsure<br>☐ Light touch ☐ Proprioception ☐ Vibration ☐ Temperature ☐ Pain ☐ Tingling/ paraesthesia<br>☐ Other- describe<br>Describe the distribution:<br>Sensory level: Yes/No/Unsure
Deep tendon reflexes (specify: normal/ reduced/ absent for each):<br>Biceps: _________Triceps: _______ Supinator: _______<br>Knee: ______ Ankle: ______ Plantar: Normal/ Absent/ Upwards
Is there associated pain? Yes/ No/ Unsure
Cranial nerve involevement:<br>Ptosis: Yes/ No/ Unsure<br>Diplopia: Yes/ No/Unsure<br>Facial weakness: Yes/ No/ Unsure<br>If yes: right/left/ bilateral If yes: LMN / UMN (forehead sparing)<br>Dysphagia/ bulbar weakness: Yes/ No/ Unsure<br>Cranial nerve involvement not captured above:
Is there shortness of breath? Yes/ No/ Unsure
Is there evidence of neuromuscular respiratory failure? Yes/No/Unsure
Is there dysautonomia? Yes/ No/ Unsure
Is there ataxia or other cerebellar features? Yes/ No/ Unsure
Other relevant symptoms and signs, including systemic features:
Has the patient experienced a similar neurological event before? Yes/No/Unsure<br>If Yes, please provide details, including the date and suspected triggers:

**Assessment and investigations to exclude other causes** (please indicate which of the following have been considered, and give details at the bottom)	
**Clinical assessment** (give details at the bottom)	
Is there:<br>▪ Suspicion of drug-induced neuropathy<br>▪ History of recent diarrhoea	 Yes/ No/ Unknown<br>Yes/ No/ Unknown
**Laboratory investigations** (if abnormal give details at the bottom)	
FBC	Normal/ Unknown/ Not Done/ Abnormal
Urea and electrolytes	Normal/ Unknown/ Not Done/ Abnormal
Calcium profile and magnesium	Normal/ Unknown/ Not Done/ Abnormal
CRP	Normal/ Unknown/ Not Done/ Abnormal
HbA1c	Normal/ Unknown/ Not Done/ Abnormal
Thyroid function tests	Normal/ Unknown/ Not Done/ Abnormal
Vasculitc screen: ESR, ANA, anti-ds DNA, SS-A (Ro), SS-B (La), ANCA, complement	Normal/ Unknown/ Not Done/ Abnormal
Protein electrophoresis	Normal/ Unknown/ Not Done/ Abnormal
B_{12}, folate	Normal/ Unknown/ Not Done/ Abnormal
Methylmalonic acid	Normal/ Unknown/ Not Done/ Abnormal
Vitamin E	Normal/ Unknown/ Not Done/ Abnormal
Angiotensin converting enzyme	Normal/ Unknown/ Not Done/ Abnormal
Serum paraneoplastic antibodies*	Normal/ Unknown/ Not Done/ Abnormal
Syphilis serology	Normal/ Unknown/ Not Done/ Abnormal

*This form, based on the guidance from the Brighton Collaboration (Sejvar et al., Vaccine 2011; 29: 599–612) and produced by the Covid-Neuro Network of the University of Liverpool's Brain Infections Global Programme, is for recording details of suspected neurological adverse events following immunisation with COVID-19 vaccines. Please feel free to modify as necessary.
Please liaise with Professor Tom Solomon and the Covid-Neuro team (Email COVIDNeuro@liverpool.ac.uk) if you are interested in contributing your data to the international collaborative study.

COVID-Neuro Network

Brain Infections Global

HIV serology<br>Lyme disease serology<br>Campylobacter jejuni serology<br>COVID-19 PCR<br>Respiratory viruses screen (swab)<br>Stool culture	Normal/ Unknown/ Not Done/ Abnormal<br>Normal/ Unknown/ Not Done/ Abnormal<br>Normal/ Unknown/ Not Done/ Abnormal<br>Normal/ Unknown/ Not Done/ Abnormal<br>Normal/ Unknown/ Not Done/ Abnormal<br>Normal/ Unknown/ Not Done/ Abnormal
*Please list the antibodies tested in the paraneoplastic panel:	
Have the antiganglioside antibodies been tested? If yes, please provide results:<br>anti-GM1/ GM2/ GD1a/ GD1b/ GQ1b<br>IgG/ IgM	
CSF Biochemistry**:** CSF Protein: ________ CSF: Serum Glucose Ratio: __________<br>CSF RCC: __________ CSF WCC: _________ CSF differential: __________ Date: ___________	
CSF Oligoclonal bands<br>CSF Cytology<br>CSF Microscopy & culture<br>CSF Virology*	Normal/ Unknown/ Not Done/ Abnormal<br>Normal/ Unknown/ Not Done/ Abnormal<br>Normal/ Unknown/ Not Done/ Abnormal<br>Normal/ Unknown/ Not Done/ Abnormal
*Please list the pathogens tested within the CSF virology panel:	
Any other relevant laboratory results:	
**Radiological studies** (if abnormal give details at the bottom)	
CT Head<br>MRI Head<br>MRI Spine	Normal/ Unknown/ Not Done/ Abnormal<br>Normal/ Unknown/ Not Done/ Abnormal<br>Normal/ Unknown/ Not Done/ Abnormal
**Nerve conduction studies**:	Demyelinating/ Axonal/ Inexcitable / Equivocal/<br>Normal/ Unkown/ Not done
**Nerve biopsy:**	Normal/ Unknown/ Not Done/ Abnormal
**Details of any abnormal findings (please provide NCS report if available):**	
**Please describe if any of the findings could explain the aetiology of the event:**	

**Treatment**	
Please write YES and provide the dates for each treatment that was started:	
IV Immunoglobulin	Date:
Plasma exchange	Date:
Steroids (IV)	Date:
Steroids (Oral)	Date:
Intubation and ventilation	Date:
Other treatments not listed above:	

**Patient Outcome**
Date information provided:
Maximum level of care required:<br>Outpatient/ Medical Inpatient/ High Dependency Unit/ Intensive Care Unit
Clinical progression:<br>Was there a NEW deterioration after 8 weeks?<br>Were there more than three treatment related fluctuations?
Patient alive at last follow-up: Yes/ No

*This form, based on the guidance from the Brighton Collaboration (Sejvar et al., Vaccine 2011; 29: 599–612) and produced by the Covid-Neuro Network of the University of Liverpool's Brain Infections Global Programme, is for recording details of suspected neurological adverse events following immunisation with COVID-19 vaccines. Please feel free to modify as necessary.
Please liaise with Professor Tom Solomon and the Covid-Neuro team (Email COVIDNeuro@liverpool.ac.uk) if you are interested in contributing your data to the international collaborative study.

COVID-Neuro Network

Brain Infections Global

If No, was GBS or neuropathy included on the death certificate: Yes/ No/ Unknown<br>If relevant, date of death:
Has this patient been discharged: Yes/ No<br>If Yes, discharge destination:<br>Usual place of residence/ Other community/ Transfer to another hospital/ Inpatient rehabilitation/<br>If yes, Duration of admission:
Modified Ranking Scale:<br>Before adverse event:_______<br>At the discharge (if not discharged, then current mRS): ______<br>GBS Disability Score at nadir:<br>GBS Disability Score at three months:
Has the patient had any further COVID-19 vaccinations since the event?<br>If yes, please provide brand, date and outcome:
Additional details:

GBS Disability Score (for reference only)	
0	Normal
1	Minor symptoms but able to run
2	Able to walk 10 m or more without assistance but unable to run
3	Able to walk 10 m across an open space with help
4	Bedridden or chairbound
5	Requiring assisted ventilation for at least part of the day
6	Dead

*This form, based on the guidance from the Brighton Collaboration (Sejvar et al., Vaccine 2011; 29: 599–612) and produced by the Covid-Neuro Network of the University of Liverpool's Brain Infections Global Programme, is for recording details of suspected neurological adverse events following immunisation with COVID-19 vaccines. Please feel free to modify as necessary.
Please liaise with Professor Tom Solomon and the Covid-Neuro team (Email COVIDNeuro@liverpool.ac.uk) if you are interested in contributing your data to the international collaborative study.
